# Population genomics reveals moderate genetic differentiation between populations of endangered Forest Musk Deer located in Shaanxi and Sichuan

**DOI:** 10.1186/s12864-022-08896-9

**Published:** 2022-09-23

**Authors:** Gang Liu, Bao-Feng Zhang, Jiang Chang, Xiao-Long Hu, Chao Li, Tin-Tao Xu, Shu-Qiang Liu, De-Fu Hu

**Affiliations:** 1 Institute of Wetland Research, Chinese Academy of Forestry, Beijing Key Laboratory of Wetland Services and Restoration, Beijing, 100091 China; 2 College of Ecology and Nature Conservation, Beijing Forestry University, Beijing, 100085 China; 3 State Key Laboratory of Environmental Criteria and Risk Assessment, Chinese Research Academy of Environmental Sciences, Beijing, 100012 China; 4 College of Animal Science and Technology, Jiangxi Agricultural University, Nanchang, 330022 China; 5 College of Plant Science, Jilin University, Changchun, 130062 China

**Keywords:** Forest Musk Deer, Population decline, Population genomics, Genetic diversity, Genetic differentiation, Gene flow

## Abstract

**Background:**

Many endangered species exist in small, genetically depauperate, or inbred populations, hence promoting genetic differentiation and reducing long-term population viability. Forest Musk Deer (*Moschus berezovskii*) has been subject to illegal hunting for hundreds of years due to the medical and commercial values of musk, resulting in a significant decline in population size. However, it is still unclear to what extent the genetic exchange and inbreeding levels are between geographically isolated populations. By using whole-genome data, we reconstructed the demographic history, evaluated genetic diversity, and characterized the population genetic structure of Forest Musk Deer from one wild population in Sichuan Province and two captive populations from two ex-situ centers in Shaanxi Province.

**Results:**

SNP calling by GATK resulted in a total of 44,008,662 SNPs. Principal component analysis (PCA), phylogenetic tree (NJ tree), ancestral component analysis (ADMIXTURE) and the ABBA-BABA test separated Sichuan and Shaanxi Forest Musk Deer as two genetic clusters, but no obvious genetic differentiation was observed between the two captive populations. The average pairwise F_ST_ value between the populations in Sichuan and Shaanxi ranged from 0.05–0.07, suggesting a low to moderate genetic differentiation. The mean heterozygous SNPs rate was 0.14% (0.11%—0.15%) for Forest Musk Deer at the genomic scale, and varied significantly among three populations (Chi-square = 1.22, *p* < 0.05, Kruskal–Wallis Test), with the Sichuan population having the lowest (0.11%). The nucleotide diversity of three populations varied significantly (*p* < 0.05, Kruskal–Wallis Test), with the Sichuan population having the lowest genetic θ_π_ (1.69 × 10^–3^).

**Conclusions:**

Genetic diversity of Forest Musk Deer was moderate at the genomic scale compared with other endangered species. Genetic differentiation between populations in Sichuan and Shaanxi may not only result from historical biogeographical factors but also be associated with contemporary human disturbances. Our findings provide scientific aid for the conservation and management of Forest Musk Deer. They can extend the proposed measures at the genomic level to apply to other musk deer species worldwide.

**Supplementary Information:**

The online version contains supplementary material available at 10.1186/s12864-022-08896-9.

## Background

Many endangered species exist in small, genetically depauperate, or inbred populations [1], hence promoting genetic differentiation and reducing long-term population viability [2, 3]. Genetic diversity is important to the breeding and long term persistence of endangered species [4, 5]. In theory, a species with a declining population size is challenged with low levels of genetic diversity and limited gene flow [6–8], inbreeding depression [9] and even high risks of extinction [10]. This means it is necessary to conduct population genetic diversity, and structure studies on endangered species; however, such evaluations are unfortunately inadequate [11–13], and genetic consequences following the disruption of gene flow still remain unknown in many cases.

Genetic diversity available for a species is determined by the genetic background of the ancestor populations, which in turn is heavily impacted by the demographic history and contemporary human disturbance [14]. Small effective population sizes due to historical bottleneck events could expose endangered species to inbreeding and loss of genetic variation [15]. Historical climatological events and geophysical barriers can alter range shifts, determine dispersal patterns and restrict gene flow, leading to significant changes in population size and genetic diversity [16]. Contemporary human-driven habitat loss and fragmentation are among the greatest threats to population decline and isolation, leading to genetic diversity loss [17, 18].

All musk deer species (*Moschus spp*.) are assessed to be globally threatened due to poaching and habitat loss [19–21], with six being ranked as endangered and one as vulnerable (*M. moschiferus*) according to The IUCN Red List of Threatened Species v. 2015 [22]. The musk deer is characterized by the musk produced by the musk pod on the abdomen of adult males, and the musk is valuable for use in traditional Chinese medicine and perfumes [23]. Consequently, musk deer species have been subject to intensive hunting for hundreds of years, resulting in severe population declines worldwide. The Forest Musk Deer (*M. berezovskii*) was once widely distributed in China [24]. However, its wild populations have been reduced dramatically due to illegal hunting and deforestation since the 1950s. Captive breeding of Forest Musk Deer has been practiced for several decades, with the aim to provide sources for the reintroduction project and help to invert population declines and hunting pressure in the wild. The population growth of Forest Musk Deer was quite slow before 2000 [25], but the last two decades have seen a relatively rapid increasing, with more than 11,340 individuals in captivity in Shaanxi Province, China (https://china.huanqiu.com/article/9CaKrnJWMYT). During the breeding, Forest Musk Deer is challenged with disease severity and immunity reduction, which may be exacerbated by inbreeding and genetic diversity declines in Forest Musk Deer [26, 27].

The development of next-generation sequencing technologies brought a new discipline “conservation genomics” into population studies of endangered species [28, 29]. Since genome-wide genetic diversity can be estimated with neutral and non-neutral genomic markers [30–33], this advantage generally leads to an unbiased estimation of genetic diversity [34]. Inbreeding estimates by the genomic data are usually less downwardly biased compared to that from traditional pedigree methods [2, 35, 36]. Genetic structure can be revealed at a relatively fine scale, even when the sign of population genetic differentiation is subtle [37]. Genomic data are also useful for reconstructing the species’ demographic history, which is essential to guide conservation activities, especially when the population is in decline [31, 38]. In aiding the project of captive breeding, as well as the reintroduction, accurate information derived from genomic data is crucial for selecting source populations to minimize inbreeding [39–41]. Previous studies mainly focused on comparing genetic diversity among captive populations of Forest Musk Deer, and depended on traditional molecular markers, including mitochondrial DNA [42, 43], microsatellites [44, 45], MHC [46, 47] and RAD sequencing [48]. The current distribution of Forest Musk Deer is limited mainly to Shaanxi and Sichuan provinces in China. however, it remains unclear to what extent the level of gene flow is between geographically isolated populations at the genomic scale. It is also unknown how demographic history and human disturbance impact the population structure.

In this study, whole genome sequencing (WGS) data were used to reconstruct the demographic history, evaluate genetic diversity, characterize genetic structure, and reveal inbreeding pattern of Forest Musk Deer in one wild population in Sichuan Province and two captive populations in Shaanxi Province, China. The current captive individuals are descendants of wild individuals captured from the natural habitats in the Qinling Mountains in Shaanxi. Therefore, we assume that the two captive populations can represent the wild population in the Qinling Mountains in Shaanxi, or at least the imprints of demographic history on the genetic background remain. We tested the hypothesis that genetic differentiation exists between populations in Sichuan and Shaanxi, and inferred the pattern of genetic composition.

## Results

### Sequencing and mapping assessment

Error rate of sequencing data was lower than 0.04% for all 15 samples of Forest Musk Deer (Additional file 1: Table S1), and the average GC content was 43.32% (Additional file 1: Table S2). After mapping to the reference genome of Forest Musk Deer, it yielded an average sequencing depth of 25 × (range: 16—30) and an average mapping percentage of 98.47% (range: 93.11%—99.55%) (Additional file 1: Table S3). The kinship coefficient was less than 0.177 for all individual pairs within each population (Additional file 1: Table S4), suggesting the genetic relatedness is not a major concern for the subsequent analysis. SNPs calling by GATK4 [49] resulted in a total of 44,008,662 SNPs after variant calling.

### Population genetic structure

**Fig. 1:**
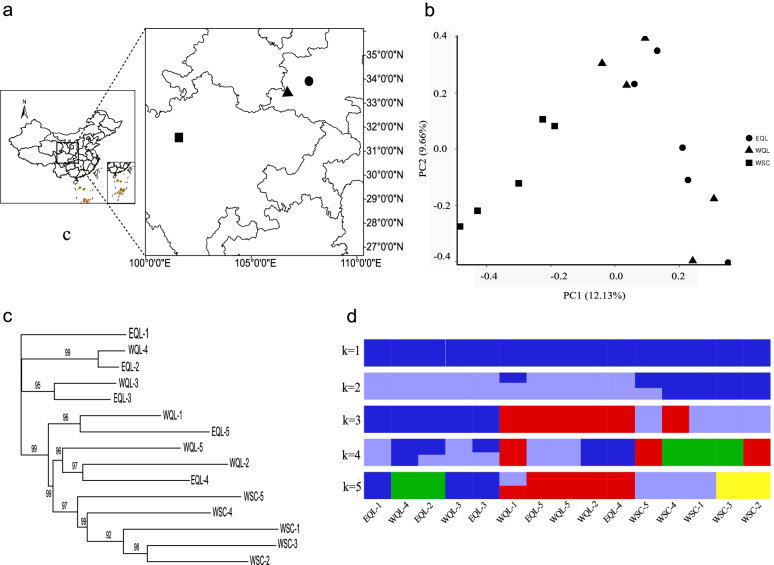
Distribution map and population genetic structure of Forest Musk Deer from WSC west Sichuan province and EQL and WQL in Shaanxi province. **a** Distribution map of Forest Musk Deer and the sampling sites in this study. **b** Top two principal component axes of genetic variation of Forest Musk Deer populations, and percentage variation explained by each principal component indicated in brackets. **c** Neighbor-joining (NJ) phylogenetic tree. **d** Subgroups represented by the ADMIXTURE analysis (k = 1, 2, 3,4 and 5 shown). The wild population, west Sichuan (WSC), and two captive populations in Shaanxi, east Qinling (EQL) and west Qinling (WQL)

For the wild population, west Sichuan (WSC), and two captive populations in Shaanxi, east Qinling (EQL) and west Qinling (WQL) (Fig. 1a), the principal component analysis (PCA, Fig. 1b) was consistent with the results of the phylogeny relationships (Fig. 1c) and the admixture algorithm (Fig. 1d). The first principal component separated among populations, and the second one indicated some within population structure, which revealed that Sichuan and Shaanxi Forest Musk Deer could be discriminated by genomic data. The first two eigenvectors explained 12.13% genetic variation, suggesting that only a subset of genetic loci played a role driving the genetic differences between populations (Fig. 1b). The Neighbor-joining (NJ) phylogenetic tree based on pairwise SNP differences revealed separate genetic clusters between populations in Shaanxi and Sichuan, but no obvious genetic differentiation was observed between the two captive populations (EQL and WQL) (Figs. 1c). In contrast, the results from ADMIXTURE confidently discriminated between two genetically distinct populations under k = 2 (Fig. 1d), although the lowest CV-error was obtained with k = 1 (Additional file 2: Figure S1). The average pairwise F_ST_ value between WSC and EQL was 0.07, 0.02 between WQL and EQL and 0.05 between WSC and WQL population, suggesting a low to moderate genetic differentiation, which was more than that between WQL and EQL in Shaanxi.

### Genetic diversity

By estimating the proportion of heterozygous SNPs per base pair, the mean heterozygous SNP rate was 0.14% (0.11%—0.15%) for Forest Musk Deer at the genomic scale, and varied significantly among three populations (Chi-square = 1.22, *p* < 0.05), with the wild WSC population having the lowest (0.11%). The nucleotide diversity of three populations varied significantly (*p* < 0.05), in contrast, WSC had the lowest genetic θ_π_ (1.69 × 10^–3^) and θ_w_ (1.59 × 10^–3^), while WQL had the highest θ_π_ (2.01 × 10^–3^) and θ_w_ (2.00 × 10^–3^) following by EQL (θ_π_: 1.98 × 10^–3^ and θ_w_: 1.94 × 10^–3^). In terms of both heterozygous SNP rate and nucleotide diversity, it could be seen that the wild population (WSC) in Sichuan had lower genetic diversity than the two captive populations in Shaanxi (EQL and WQL).

### Demographic history and gene flow

**Fig. 2:**
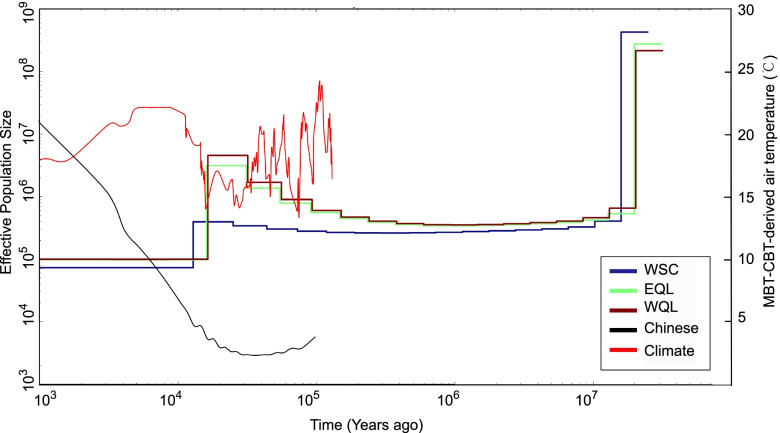
Demographic history of Forest Musk Deer reconstructed from whole genome sequencing data using the multiple sequential Markovian coalescence (MSMC) model. Inferred fluctuations in effective population size (Ne) based on the generation of 5 years and the per site per generation mutation rate of 2.2 × 10^–9^. Blue line represents estimated Ne of WSC, purple and green lines indicate EQL and WQL, respectively. Red line represents Chinese Han population (Chinese). Black line shows air temperature record reconstructed from the cyclization and methylation indexof branched tetraethers (MBT-CBT)

**Fig. 3:**
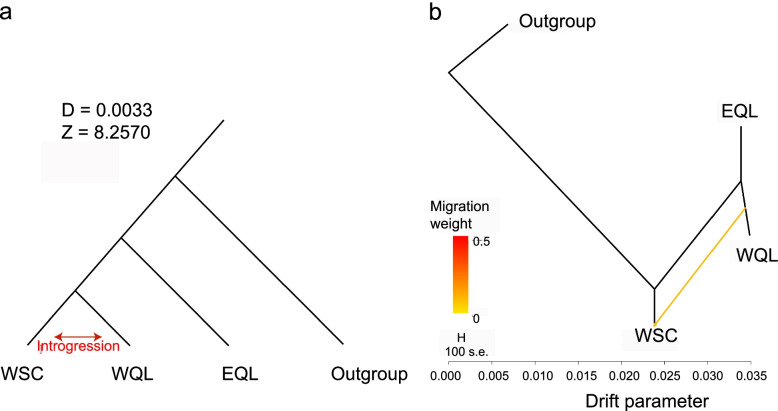
Genetic introgression and gene flow among WSC, WQL and EQL. **a** The ABBA-BABA test indicated more shared alleles between WSC and WQL. **b** Population split and historical mixture for Forest Musk Deer in the presence of Siberian Musk Deer. Arrows indicate migration events between WSC and WQL. A spectrum of heat colors indicates different migration weights at the migration event

We reconstructed historical effective populations sizes (Ne) for all Forest Musk Deer populations using the MSMC [50]. All populations show a steady population increase followed by a dramatic expansion approximately 100,000 years ago, and then the population started to decline and showed a sharp population reduction around 10,000 years ago (Fig. 2). The effective population size of WSC was lower than both WQL and EQL, while WQL and EQL showed a similar pattern. The ABBA-BABA test [51] indicated more share-derived alleles between WQL and EQL (D = 0.0033, Z = 8.257, Fig. 3a and Additional file 1: Table. S5 and S6), which suggested that WSC was genetically far from both of WQL and EQL. The tree mix result (Fig. 3b) was consistent with the ABBA-BABA test.

### Inbreeding levels

**Fig. 4:**
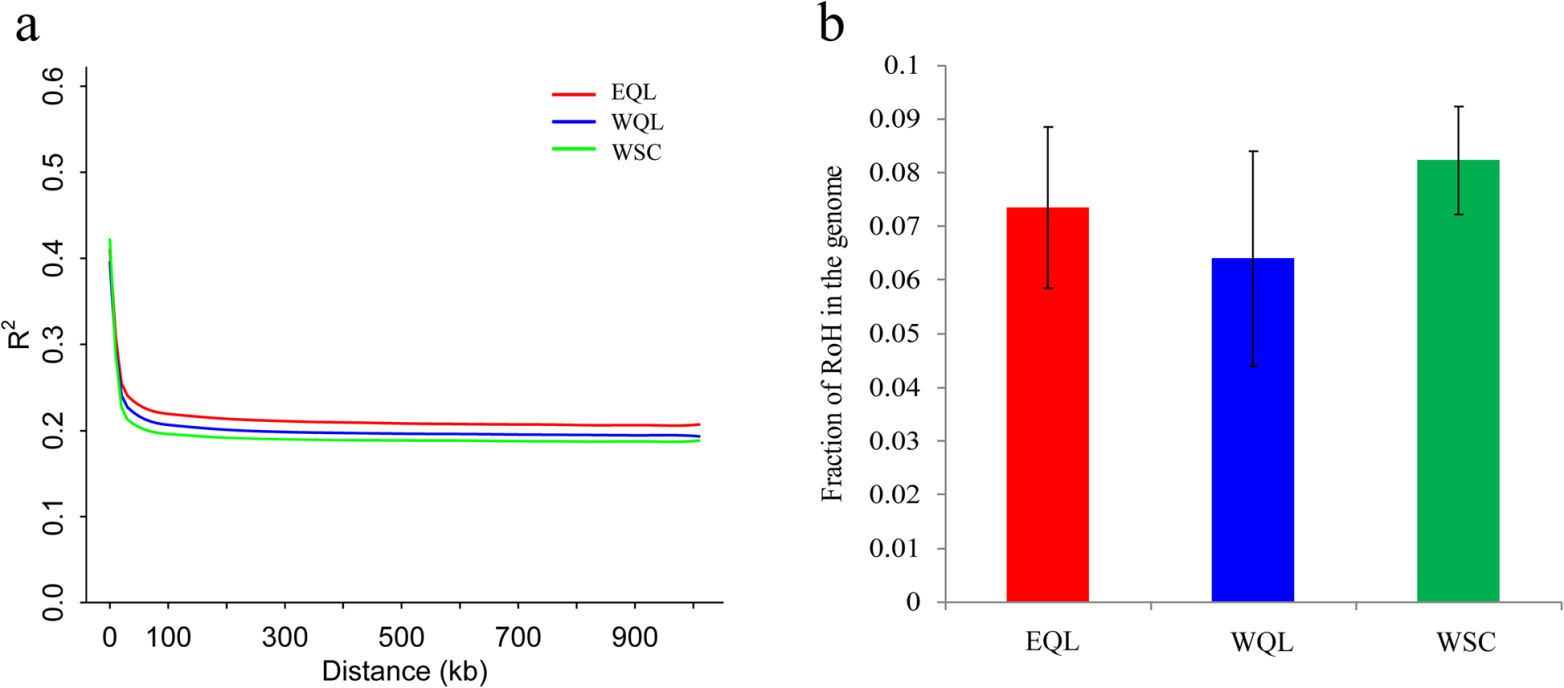
Decay of Linkage disequilibrium (LD) decay and fraction of runs of homozygosity (RoHs) in the genome. **a** LD decay is represented by change in average squared correlation coefficient (r.^2^) between SNPs among all individuals per population as physical distance increases between SNPs. **b** fraction of runs of homozygosity (RoHs)

Based on the linkage disequilibrium (LD) analysis, the extent of LD in both populations of WQL and EQL appeared consistently, but was quite different from the pattern in WSC. LD decayed to *r*^2^ < 0.2 within 10 kb, but declined more slowly (Fig. 4a). Consistently with the LD results above, the two populations of WQL and EQL had lower numbers of RoHs above 200 kb than those of WSC (Fig. 4b), but no significant difference was detected (Chi-square = 0.681, *p* > 0.05).

## Discussion

We found that the genetic diversity of the wild population in west Sichuan (WSC) was lower than that of both captive populations in Shaanxi (WQL and EQL), which was consistent with previous studies revealed by microsatellites [52], mitochondrial DNA [43], MHC [46], and RAD sequencing [48]. The highest level in Shaanxi is mainly due to abundant sources from different places, because the centers have a relatively short history and more widely distributed individuals are introduced into the breeding [52]. We compared the genome-wide heterozygosity of Forest Musk Deer with that of other mammal species (Additional file 2: Figure S2), and showed that it was higher than those in the Przewalski's Horse (0.039%) and Père David's Deer (0.054%), both of which went extinct in the wild in the early 1900s and were restored by limited founders in captivity [53]. However, the genome-wide heterozygosity of Forest Musk Deer was comparable to that of Giant panda, Chinese Pangolin and Sumatran Rhinoceros [54, 55] (Additional file 2: Figure S2). Meanwhile, the genome-wide heterozygosity of Forest Musk Deer was higher than the average (0.07%) for the mammals listed as EN in the IUCN Red List [22], suggesting that moderate genetic diversity for this species was observed. Such moderate levels of genetic diversity suggest that adaptive potential of this species still exists but is likely to decrease. The genomic consequences of long-term population declines include extreme reduction of genetic diversity and evidence of inbreeding depression [28]. However, dramatic short-term population declines need not necessarily result in major losses of genetic diversity [56].

The phylogeographic reconstruction, PCA and ADMIXTURE analyses provided evidence of moderate population genetic differentiation between populations of Forest Musk Deer located in Shaanxi and Sichuan, but weak genetic differentiation between two captive populations within Shaanxi. The distribution of wild Forest Musk Deer is divided into small populations by geographical barriers, such as Qinling Mountains and Minjiang River [57, 58], and this spatial separation may contribute to the genetic differentiation observed in Sichuan and Shaanxi. Past climate change is considered to be main drivers to shape the demographic history and genetic structure of terrestrial mammals [16]. For example, the geographical separation of the Qinling Mountains (Shaanxi) from the Minshan mountains (Sichuan) has caused morphological, behavioral and genetic distinctions of Giant Panda (*Ailuropoda melanoleuca*) [59, 60]. Cooling period during the Pleistocene may act on genetic differentiation of Forest Musk Deer similarly to Giant Panda by influencing gene flow between Sichuan and Shaanxi. A sudden rise in air temperature after the Last Glacial Maximum (LGM) about 12 kya might have change the forest habitats, and in turn might have driven the separation of Forest Musk Deer. In addition, the Sichuan Basin is not the refuge for Forest Musk Deer during the last glaciations, since Forest Musk Deer is a typical ruminant that prefers the high-altitude environment, instead of the warm and humid climate in the basin, which implies that the Sichuan Basin may also pose a geographical barrier to gene flow. Similar origin of location and individual exchange between ex-situ centers within Shaanxi would help to explain the observed low genetic differentiation between the two captive populations (WQL and EQL).

It is important to note that most mammals contract during the most recent glacial period approximately 12.1 – 34.8 kya [59]. Forest Musk Deer population did not recover even after the temperature trajectory reversed, which suggested that, besides the biogeographical factors, human activities may have contributed to accelerating the population differentiation and decline. The distribution of wild Forest Musk Deer is characterized by scattered habitats being lost due to deforestation in the past and isolated by towns, roads, railways, and other infrastructure, which exacerbates the effects of habitat fragmentation and consequently restricts gene flow.

The demographic history based on genomic data further provided evidence that human disturbance was closely associated with the population declines of Forest Musk Deer. It could be inferred that the effective population size of Forest Musk Deer was impacted by human activities because the Chinese Han population continued to increase since 30 kya, whereas the effective population size experienced a steep decline in Forest Musk Deer. Forest Musk Deer, a species that adapts to high mountain forests and is characterized with shy and timid personality, is more vulnerable to the natural landscape changes and the destruction of hidden conditions [61]. Dramatic population decline due to human overexploitation, such as deforestation, poaching and harvest, can influence reproduction and survival rates, and lead to extremely low population density (0.16–0.24 individuals per km^2^ [62]).

Musk has been used for perfume and medicinal purposes for thousands of years Unfortunately for the endangered but economically important species, the species conservation and musk utilization are unbalanced. Current conservation efforts remain insufficient to offset the negative effects of population declines. The conservation priority is to enhance the patrol time to eliminate poaching [63, 64], which could eventually help restore the population and maintain the genetic diversity. In order to ensure population persistence, we propose additional conservation measures based on our findings. First, effective measures should be carried out to increase wild populations and restore suitable habitats, because targeted gene flow can allow purifying selection to function well and effectively to purge deleterious mutations. Targeted gene flow indicates translocating individuals with rare or favorable genes to areas. Second, the populations of Forest Musk Deer in Sichuan and Shaanxi are suggested to be considered as independent conservation management units. Third, artificial gene flow among different ex-situ centers should be prompted, and individuals with higher genetic diversity can be the candidate to be released into the wild.

The effects of recent severe demographic and genetic declines can be inferred by comparing the contemporary samples with the historical samples, or by running simulations under alternative historical scenarios based on the coalescent approach. This will be the research priority to evaluate the genetic consequences of wildlife commercial and medical exploitation within a comparative context. For captive populations, breeding management is an important factor in determining the genetic composition and population genetic structure, which means that pedigree records are strongly suggested to be done in order to avoid inbreeding in captivity. The proposed measures at the genomic level could be extended to apply to other musk deer species worldwide, since the knowledge from population genomics will contribute to the conservation of those endangered species.

## Methods

### DNA sample collection

Tissue or blood samples of 15 Forest Musk Deer individuals were collected from one wild population and two ex-situ populations. Specifically, five skin tissue samples were collected from the wild individuals captured in the west of Sichuan (WSC), five blood samples from the ex-situ center in Fengxian located in the west of Qinling Mountains (WQL) and five blood samples from the ex-situ center in Meixian located in the east of Qinling Mountains (EQL) (Fig. 1a). The WQL was built in 1986, with a current population size of 258. The EQL was constructed in 2003 and has 196 individuals by the end of 2020. Both the WQL and EQL populations are composed of descendants of wild individuals from their natural habitats in the Qinling Mountains, and the number of founders is not known due to a lack of pedigree records. The exchange of captive-bred individuals occur less frequently among different ex-situ centers when captive-bred within Shaanxi Province. Blood samples were stored in vacuum blood collection tube (EDTA anticoagulation), and tissue samples were preserved using ethanol. All samples were stored at -80 ℃ (Additional file 1: Table S1).

### Genome sequencing and SNP calling

Genomic DNA was extracted using QIAamp DNA Blood Mini Kit (Qiagen, Germany). The libraries were prepared as described in the commercial TruSeq kit (Illumina, the U.S.A). Whole-genome paired-end sequencing (PE150) was performed within the Illumina Novaseq 6000 platform using standard procedures (Novogen, China). The raw FASTQ data was de-multiplexed, and the adapters were trimmed using Trimmomatic v0.36.0 [64]. Clean sequencing reads were aligned against the reference genome of Forest Musk Deer downloaded from NCBI (BioSample ID: SAMN10822789, BioProject ID: PRJNA438286) using Burrows-Wheeler Alignment (BWA v0.7) tool [65]. Read alignments were sorted using SAMTools v1.11 [65], and duplicate reads were marked by Picard.

Variant calling was conducted using HaplotypeCaller and GenotypeGVCFs modules in GATK4 [49]. To ensure variant identification accuracy, GATK best practices including the density distribution of each parameter and the filtering criteria. SNPs were removed if any of the following parameter was met: missingness > 0.9, minor allele frequency < 0.05, QD < 2, MQ < 30, MQRankSum <  − 12.5, FS > 200, ReadPosRankSum < 20, QUAL < 30.0, AN < 40. The GVCF file from each sample was generated using HaplotypeCaller, and then GenotypeGVCFs was used to merge separate GVCF files with the aim of improving the sensitivity of variant detection. Before performing the subsequent population genomics analyses, genetic relatedness between individuals within each population was calculated using GCTA v1.94.0 [66]. Individual pairs were excluded from the subsequent analyses if their kinship coefficient was greater than 0.177, a threshold showing twins or first-degree relationships [67].

### Genetic diversity estimation

In order to assess genetic diversity within and among Forest Musk Deer populations, the heterozygosity of each individual was calculated by dividing the total number of heterozygous SNPs by genome size as previously described and averaged over each population [68]. Population genetics statistics θ_π_ was calculated using VCFtools v0.1.13 [69]_,_ and the Watterson’s estimator θ_w_ was calculated using VariScan v2.0.3 [70].

### Population structure analysis

The population structure analysis was performed using the commonly used methods [71], including phylogenetic tree (NJ tree), ancestral component analysis and principal component analysis (PCA). Based on the filtered SNPs, the GCTA program v1.94.0 [66] was applied to conduct PCA, by filtering the principal components through an algorithm of dimensionality reduction. The NJ tree was built using TreeBeST v1.9.2 (http://treesoft.sourceforge.net/) with the aim to visualize the genetic distance relationship between individuals, with a bootstrap value of 1,000. The standard VCF file was filtered and thinned according to user-specified command line options (–maf 0.05 –max-missing 0.9 –min-alleles 2 –max-alleles 2) by VCFtools v0.1.13 [69]. Additional filtering was handled for LD pruning (–indep-pairwise 50 5 0.2) using PLINK v.1.9 [72], and then population genetic structure was inferred using ADMIXTURE v1.3.0 [73]. The most likely value of K was identified from 20 independent runs for each value of K ranging from two to five, with 100 bootstraps. We used VCFtools v0.1.13 [69] to calculate pairwise Fst, a widely used genetic differentiation statistics, to examine the genetic differentiation between the three Forest Musk Deer populations (WSC, WQL, and EQL).

### Demographic history reconstruction

Multiple sequential Markovian coalescence (MSMC) model can be used to reconstruct each population’s demographic history based on genomic information [50]. MSMC is developed to overcome the limitation that the pairwise sequentially Markovian coalescent (PSMC) can only analyze one sample at a time [74]. In addition, MSMC can integrate and analyze the nearest common ancestor time between multiple allele sequences at the same time [75], thereby improving the accuracy and efficiency of effective population size (Ne) prediction. We performed demography inference using msmc2 v2.1.2 [75]. The parameters used were as follows: “-t 6 -p 1*2 + 15*1 + 1*2”. A mutation rate (*μ*)　of 2.2 × 10^–9^ mutations per site per year for Forest Musk Deer used in this study was according to the reference [17]. The generation time was estimated to be five years based on a report of mammal generation length [76].

### Gene flow analysis

The Patterson's D-statistic, also called ABBA-BABA test, is the most widely used statistical approach that is applied to detect introgression across genomes between.

populations within a species or among species [51], and reveal the genomic footprint of post speciation introgression [77]. The appeal of D statistics is its simplicity, but the timing and direction of introgression can not be inferred [78]. We downloaded the whole genome re-sequencing data of Siberian Musk Deer (*M. moschiferus*) from NCBI (Project ID: PRJNA574937), and used it as the outgroup. We performed the test using angsd v0.935 [79], under the order set as WSC/WQL/EQL/outgroup or WSC/EQL/WQL/outgroup. The commands were as the following: angsd -doAbbababa2 1 -rmTrans 0 -blockSize 5,000,000 -doCounts 1 -enhance 1 -maxDepth 100. The D statistic was estimated using the formula [50], and z scores were calculated using all samples of each population. To test for significance, *p*-values were computed based on a two-tailed binomial test. Meanwhile, inference of gene flow was conducted based on genome-wide allele frequency data using the software TreeMix v1.12 [80].

### Detection of inbreeding patterns

Detection of linkage disequilibrium (LD) patterns is necessary to infer historical changes of population and inbreeding patterns in the species demographic history [81, 82]. To assess the genomic extent of inbreeding in Forest Musk Deer, genome-wide LD was estimated for the three populations using PopLDdecay v1.0 [83]. The squared correlation coefficient (r^2^) between any two loci from each population was calculated using VCFtools v0.1.13 [69], with the following parameters: “–ld -window -bp 500,000 -geno -r2”, and average *r*^2^ values were identified for pairwise SNPs by keeping the same distance.

To detect the genomic signatures of inbreeding in Forest Musk Deer, genome-wide runs of homozygosity (RoHs) was identified for each individual using the tool in PLINK v.1.9 [72], with the following parameters (-threshold 0.05 -snp 65 -kb 100 -missing 3 -gap 5,000 -density 5,000).

## Supplementary Information

**Additional file 1: Table S1.** The sample information of 15 Forest Musk Deer individuals and the BioProject accession number to retrive raw data from the NCBI website is PRJNA765065. **Table S2.** Raw reads, sequencing error rate and GC content of 15 Forest Musk Deer samples. **Table S3.** Mapping and coverage statistics for 15 samples of Forest Musk Deer. **Table S4.** The kinship coefficient between individuals. The values below the diagonal refer to the kinship coefficient for all individual pairs. **Table S5.** The result of ABBA-BABA test results based on the Observe. Table S6. The result of ABBA-BABA test results based on the TransRem.

**Additional file 2: Figure S1.** Cross validation error (CV) plot from ADMIXTURE. **Figure S2.** Comparison of the genome-wide heterozygosity among species under different conservation status. (a) Crossoptilon mantchuricum exhibits lowest level of genetic diversity among bird species based on available estimates from genome-wide sequencing data. Genome-wide heterozygosity was a useful indicator of genetic diversity, which was measured as proportion of heterozygous SNPs per base pair and plotted in rank order for 91 mammal species including Forest musk deer. Colored bars indicate the IUCN Red List Categories and Criteria (https://www.iucnredlist.org/). References for each species were listed and provided in the supplementary dataset. Data of 89 mammal species except Forest musk deer and Père David's Deer were from the supplementary file in Hu JY, Hao ZQ, Frantz L, Wu SF, Chen W, Jiang YF, Wu H, Kuang WM, Li H, Zhang YP et al: Genomic consequences of population decline in critically endangered pangolins and their demographic histories. Natl Sci Rev 2020, 7(4):798-814.

## Data Availability

The whole genome sequencing data for all 15 individuals generated in this study have been deposited at NCBI SRA Database with BioProject accession ID: PRJNA765065. The accession number for each sample of Forest musk deer can be seen in the Additional file 1: Table S1.

## Abbreviations

QL: Qinling
SC: Sichuan
MHC: Major Histocompatibility Complex
RAD: Restriction association site DNA
BAM: Binary Alignment
NCBI: National Center for Biotechnology Information
BWA: BurrowsWheeler Aligner
F_ST_: Fixation index
LD: Linkage disequilibrium
GATK: Genome Analysis Toolkit
θπ: Nucleotide diversity
PCA: Principle component analysis
RoHs: Runs of homozygosity
SNP: Single-nucleotide polymorphism
LGM: Last Glacial Maximum
kya: 1,000 Years ago
MBT-CBT: Cyclization and methylation indexof branched tetraethers
