# Supplementary material for: Population genomics reveals moderate genetic differentiation between populations of endangered Forest Musk Deer located in Shaanxi and Sichuan
Source: BMC Genomics. 2022 Sep 23;23:668. doi: 10.1186/s12864-022-08896-9 (PMC9503231; doi:10.1186/s12864-022-08896-9)
Supplement: Supplementary file 1 — Additional file 1: Table S1. The sample information of 15 Forest Musk Deer individuals and the BioProject accession number to retrive raw data from the NCBI website is PRJNA765065. Table S2. Raw reads, sequencing error rate and GC content of 15 Forest Musk Deer samples. Table S3. Mapping and coverage statistics for 15 samples of Forest Musk Deer. Table S4. The kinship coefficient between individuals. The values below the diagonal refer to the kinship coefficient for all individual pairs. Table S5. The result of ABBA-BABA test results based on the Observe. Table S6. The result of ABBA-BABA test results based on the TransRem. [file 12864_2022_8896_MOESM1_ESM.docx]

**Additional file 1**

Table S1. The sample information of 15 Forest Musk Deer individuals and the BioProject accession number to retrive raw data from the NCBI website is PRJNA765065.

| **Sample** | **Population** | **Sample type** | **Collection time** | **Sampling site** | **SRA accession number** |
| --- | --- | --- | --- | --- | --- |
| EQL-1 | EQL | Blood | June 2018 | ex situ center in Meixian, Shaanxi Province | SRR19913423 |
| EQL-2 | EQL | Blood | June 2018 | ex situ center in Meixian, Shaanxi Province | SRR19913422 |
| EQL-3 | EQL | Blood | June 2018 | ex situ center in Meixian, Shaanxi Province | SRR19913421 |
| EQL-4 | EQL | Blood | June 2018 | ex situ center in Meixian, Shaanxi Province | SRR19913420 |
| EQL-5 | EQL | Blood | June 2018 | ex situ center in Meixian, Shaanxi Province | SRR19913419 |
| WQL-1 | WQL | Blood | July 2018 | ex situ center in Fengxian, Shaanxi Province | SRR19913418 |
| WQL-2 | WQL | Blood | July 2018 | ex situ center in Fengxian, Shaanxi Province | SRR19913417 |
| WQL-3 | WQL | Blood | July 2018 | ex situ center in Fengxian, Shaanxi Province | SRR19913416 |
| WQL-4 | WQL | Blood | July 2018 | ex situ center in Fengxian, Shaanxi Province | SRR19913425 |
| WQL-5 | WQL | Blood | July 2018 | ex situ center in Fengxian, Shaanxi Province | SRR19913424 |
| WSC-1 | WSC | Tissue | Nov 2019 | the wild from the west of Sichuan | SRR16002985 |
| WSC-2 | WSC | Tissue | Nov 2019 | the wild from the west of Sichuan | SRR16002984 |
| WSC-3 | WSC | Tissue | Nov 2019 | the wild from the west of Sichuan | SRR16002983 |
| WSC-4 | WSC | Tissue | Nov 2019 | the wild from the west of Sichuan | SRR16002982 |
| WSC-5 | WSC | Tissue | Nov 2019 | the wild from the west of Sichuan | SRR16002981 |

Table S2. Raw reads, sequencing error rate and GC content of 15 Forest Musk Deer samples

| Sample | Raw Reads | Clean Reads | Raw Base(G) | Clean Base(G) | Effective Rate(%) | Error Rate(%) | Q20(%) | Q30(%) | GC Content(%) |
| --- | --- | --- | --- | --- | --- | --- | --- | --- | --- |
| EQL-1 | 330,861,421 | 329,488,589 | 99.26 | 98.85 | 99.59 | 0.04 | 95.55 | 88.77 | 42.91 |
| EQL-2 | 246,492,446 | 245,464,394 | 73.95 | 73.64 | 99.58 | 0.03 | 96.34 | 90.44 | 43.05 |
| EQL-3 | 317,767,746 | 316,508,978 | 95.33 | 94.95 | 99.60 | 0.03 | 95.96 | 89.56 | 42.82 |
| EQL-4 | 338,202,212 | 336,912,040 | 101.46 | 101.07 | 99.62 | 0.03 | 96.53 | 90.88 | 42.64 |
| EQL-5 | 319,843,528 | 318,620,239 | 95.95 | 95.59 | 99.62 | 0.03 | 96.59 | 91.00 | 42.89 |
| WQL-1 | 302,327,852 | 301,152,065 | 90.7 | 90.35 | 99.61 | 0.03 | 96.41 | 90.57 | 42.98 |
| WQL-1 | 299,319,709 | 298,036,974 | 89.8 | 89.41 | 99.57 | 0.04 | 95.55 | 88.84 | 42.91 |
| WQL-1 | 293,506,636 | 292,090,911 | 88.05 | 87.63 | 99.52 | 0.04 | 94.88 | 87.55 | 42.93 |
| WQL-1 | 317,201,913 | 315,929,609 | 95.16 | 94.78 | 99.60 | 0.03 | 96.80 | 91.44 | 42.79 |
| WQL-1 | 341,390,120 | 340,041,449 | 102.42 | 102.01 | 99.60 | 0.04 | 95.56 | 88.78 | 43.11 |
| WSC-1 | 230,870,796 | 229,510,954 | 69.26 | 68.85 | 99.41 | 0.03 | 97.41 | 93.01 | 45.91 |
| WSC-2 | 202,869,059 | 202,089,274 | 60.86 | 60.63 | 99.62 | 0.03 | 97.00 | 92.04 | 43.32 |
| WSC-3 | 217,203,094 | 216,149,230 | 65.16 | 64.84 | 99.51 | 0.03 | 97.26 | 92.60 | 44.50 |
| WSC-4 | 204,023,496 | 203,019,868 | 61.21 | 60.91 | 99.51 | 0.03 | 97.03 | 92.06 | 43.47 |
| WSC-5 | 204,246,644 | 203,274,327 | 61.27 | 60.98 | 99.52 | 0.03 | 96.88 | 91.83 | 43.58 |

Table S3. Mapping and coverage statistics for 15 samples of Forest Musk Deer.

| Sample | Clean_reads | mapped_reads | mapping_rate | Average_depth | Coverage_1X | Coverage_4X |
| --- | --- | --- | --- | --- | --- | --- |
| EQL-1 | 6.37E+08 | 6.34E+08 | 99.53% | 27.78 | 94.72% | 94.10% |
| EQL-2 | 4.07E+08 | 4.04E+08 | 99.36% | 18.38 | 94.58% | 93.29% |
| EQL-3 | 4.06E+08 | 3.78E+08 | 93.11% | 17.4 | 94.58% | 93.20% |
| EQL-4 | 4.04E+08 | 3.95E+08 | 97.78% | 17.83 | 94.57% | 93.25% |
| EQL-5 | 4.59E+08 | 4.29E+08 | 93.46% | 16.08 | 94.12% | 87.08% |
| WQL-1 | 4.32E+08 | 4.25E+08 | 98.36% | 18.67 | 94.59% | 92.95% |
| WQL-1 | 5.84E+08 | 5.81E+08 | 99.39% | 27 | 94.76% | 94.12% |
| WQL-1 | 6.74E+08 | 6.71E+08 | 99.53% | 29.51 | 94.74% | 94.15% |
| WQL-1 | 6.33E+08 | 6.3E+08 | 99.50% | 28.94 | 94.85% | 94.29% |
| WQL-1 | 4.91E+08 | 4.89E+08 | 99.51% | 21.88 | 94.66% | 93.80% |
| WSC-1 | 6.59E+08 | 6.55E+08 | 99.46% | 30.11 | 94.73% | 94.15% |
| WSC-2 | 6.02E+08 | 6E+08 | 99.55% | 26.58 | 94.72% | 94.07% |
| WSC-3 | 5.96E+08 | 5.93E+08 | 99.45% | 27.37 | 94.70% | 94.05% |
| WSC-4 | 6.32E+08 | 6.29E+08 | 99.55% | 28.22 | 94.74% | 94.12% |
| WSC-5 | 6.8E+08 | 6.77E+08 | 99.48% | 30.54 | 94.75% | 94.17% |

Table S4 The kinship coefficient between individuals. The values below the diagonal refer to the kinship coefficient for all individual pairs.

|  | WSC_1 | WSC_2 | WSC_3 | WSC_4 | WSC_5 | EQL_5 | EQL_4 | EQL_3 | EQL_2 | EQL_1 | WQL_1 | WQL_3 | WQL_2 | WQL_4 | WQL_5 |
| --- | --- | --- | --- | --- | --- | --- | --- | --- | --- | --- | --- | --- | --- | --- | --- |
| WSC_1 |  |  |  |  |  |  |  |  |  |  |  |  |  |  |  |
| WSC_2 | 0.113 |  |  |  |  |  |  |  |  |  |  |  |  |  |  |
| WSC_3 | 0.129 | 0.206 |  |  |  |  |  |  |  |  |  |  |  |  |  |
| WSC_4 | 0.060 | 0.059 | 0.056 |  |  |  |  |  |  |  |  |  |  |  |  |
| WSC_5 | 0.051 | 0.042 | 0.036 | 0.059 |  |  |  |  |  |  |  |  |  |  |  |
| EQL_5 | -0.005 | -0.011 | -0.015 | 0.003 | 0.007 |  |  |  |  |  |  |  |  |  |  |
| EQL_4 | 0.013 | 0.010 | 0.005 | 0.024 | 0.033 | 0.082 |  |  |  |  |  |  |  |  |  |
| EQL_3 | -0.004 | -0.010 | -0.012 | 0.025 | 0.008 | 0.056 | 0.051 |  |  |  |  |  |  |  |  |
| EQL_2 | -0.021 | -0.023 | -0.027 | -0.015 | -0.004 | 0.062 | 0.033 | 0.063 |  |  |  |  |  |  |  |
| EQL_1 | -0.001 | -0.006 | -0.008 | 0.013 | 0.015 | 0.095 | 0.035 | 0.039 | 0.140 |  |  |  |  |  |  |
| WQL_1 | 0.016 | 0.009 | 0.005 | 0.120 | 0.057 | 0.147 | 0.031 | 0.034 | 0.020 | 0.045 |  |  |  |  |  |
| WQL_3 | -0.017 | -0.017 | -0.023 | -0.005 | 0.003 | 0.032 | 0.032 | 0.220 | 0.149 | 0.162 | 0.007 |  |  |  |  |
| WQL_2 | -0.001 | -0.003 | -0.011 | 0.023 | 0.019 | 0.042 | 0.121 | 0.102 | 0.016 | 0.044 | 0.039 | 0.048 |  |  |  |
| WQL_4 | 0.006 | -0.001 | -0.002 | 0.010 | 0.024 | 0.011 | 0.058 | 0.062 | 0.310 | 0.051 | 0.021 | 0.057 | 0.010 |  |  |
| WQL_5 | 0.016 | 0.013 | 0.008 | 0.025 | 0.041 | 0.081 | 0.063 | 0.040 | 0.028 | 0.038 | 0.049 | 0.047 | 0.097 | 0.044 |  |

Table S5. The result of ABBA-BABA test results based on the Observe.

| D | JK-D | V(JK-D) | Z | pvalue | nABBA | nBABA | nBlocks | H1 | H2 | H3 | H4 |
| --- | --- | --- | --- | --- | --- | --- | --- | --- | --- | --- | --- |
| 0.002902 | 0.002902 | 0 | 8.799537 | 0 | 1078745 | 1072504 | 59395 | Population_1 | Population_2 | Population_3 | Population_4 |
| -0.17572 | -0.17572 | 0 | -370.733 | 0 | 1078745 | 1538669 | 61339 | Population_1 | Population_3 | Population_2 | Population_4 |
| -0.17853 | -0.17853 | 0 | -370.261 | 0 | 1072504 | 1538669 | 61148 | Population_2 | Population_3 | Population_1 | Population_4 |

Table S6. The result of ABBA-BABA test results based on the TransRem.

| D | JK-D | V(JK-D) | Z | pvalue | nABBA | nBABA | nBlocks | H1 | H2 | H3 | H4 |
| --- | --- | --- | --- | --- | --- | --- | --- | --- | --- | --- | --- |
| 0.003362 | 0.003362 | 0 | 8.257022 | 0 | 348688.3 | 346351.6 | 49472 | Population_1 | Population_2 | Population_3 | Population_4 |
| -0.17424 | -0.17424 | 0 | -309.437 | 0 | 348688.3 | 495833.3 | 52671 | Population_1 | Population_3 | Population_2 | Population_4 |
| -0.17749 | -0.17749 | 0 | -311.118 | 0 | 346351.6 | 495833.3 | 52310 | Population_2 | Population_3 | Population_1 | Population_4 |
