# Supplementary material for: Population genomics reveals moderate genetic differentiation between populations of endangered Forest Musk Deer located in Shaanxi and Sichuan
Source: BMC Genomics. 2022 Sep 23;23:668. doi: 10.1186/s12864-022-08896-9 (PMC9503231; doi:10.1186/s12864-022-08896-9)
Supplement: Supplementary file 2 — Additional file 2: Figure S1. Cross validation error (CV) plot from ADMIXTURE. Figure S2. Comparison of the genome-wide heterozygosity among species under different conservation status. (a) Crossoptilon mantchuricum exhibits lowest level of genetic diversity among bird species based on available estimates from genome-wide sequencing data. Genome-wide heterozygosity was a useful indicator of genetic diversity, which was measured as proportion of heterozygous SNPs per base pair and plotted in rank order for 91 mammal species including Forest musk deer. Colored bars indicate the IUCN Red List Categories and Criteria (https://www.iucnredlist.org/). References for each species were listed and provided in the supplementary dataset. Data of 89 mammal species except Forest musk deer and Père David's Deer were from the supplementary file in Hu JY, Hao ZQ, Frantz L, Wu SF, Chen W, Jiang YF, Wu H, Kuang WM, Li H, Zhang YP et al: Genomic consequences of population decline in critically endangered pangolins and their demographic histories. Natl Sci Rev 2020, 7(4):798-814. [file 12864_2022_8896_MOESM2_ESM.docx]

**Additional file 2**

**Figure S1.** Cross validation error (CV) plot from ADMIXTURE.

**
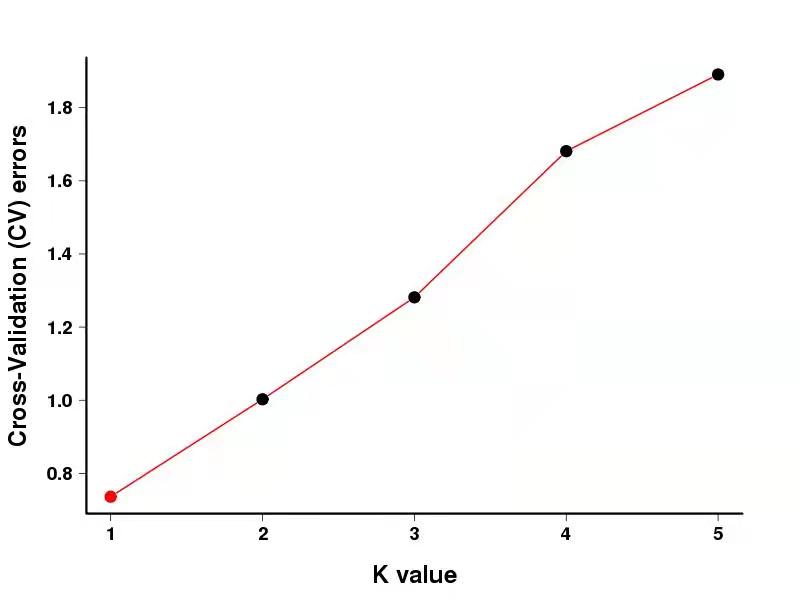
**

**Figure S2.** Comparison of the genome-wide heterozygosity among species under different conservation status. (a) Crossoptilon mantchuricum exhibits lowest level of genetic diversity among bird species based on available estimates from genome-wide sequencing data. Genome-wide heterozygosity was a useful indicator of genetic diversity, which was measured as proportion of heterozygous SNPs per base pair and plotted in rank order for 91 mammal species including Forest musk deer. Colored bars indicate the IUCN Red List Categories and Criteria (https://www.iucnredlist.org/). References for each species were listed and provided in the supplementary dataset. Data of 89 mammal species except Forest musk deer and Père David's Deer were from the supplementary file in Hu JY, Hao ZQ, Frantz L, Wu SF, Chen W, Jiang YF, Wu H, Kuang WM, Li H, Zhang YP et al: Genomic consequences of population decline in critically endangered pangolins and their demographic histories. Natl Sci Rev 2020, 7(4):798-814.
